# Mutant KRAS and GATA6 Stratify Survival in Patients Treated with Chemotherapy for Pancreatic Adenocarcinoma: A Prospective Cohort Study

**DOI:** 10.3390/cancers17050896

**Published:** 2025-03-05

**Authors:** Jung Won Chun, Dong-eun Lee, Nayoung Han, SooBeen Heo, Hyeji Kim, Mi Rim Lee, Hyeong Min Park, Sung-Sik Han, Sang-Jae Park, Tae Hyun Kim, Woo Jin Lee, Yun-Hee Kim, Sun-Young Kong, Sang Myung Woo

**Affiliations:** 1 Center for Liver and Pancreatobiliary Cancer, National Cancer Center, 323 Ilsan-ro, Ilsandong-gu, Goyang-si 10408, Republic of Korea; 2 Research Institute, National Cancer Center, 323 Ilsan-ro, Ilsandong-gu, Goyang-si 10408, Republic of Korea; 3 Cancer Biomedical Science, National Cancer Center, Graduate School of Cancer Science and Policy, 323 Ilsan-ro, Ilsandong-gu, Goyang-si 10408, Republic of Korea; 4 Department of Pathology, National Cancer Center, 323 Ilsan-ro, Ilsandong-gu, Goyang-si 10408, Republic of Korea; 5 Targeted Therapy Branch, Center for Rare Cancers, National Cancer Center, 323 Ilsan-ro, Ilsandong-gu, Goyang-si 10408, Republic of Korea; 6 Department of Laboratory Medicine, National Cancer Center, 323 Ilsan-ro, Ilsandong-gu, Goyang-si 10408, Republic of Korea

**Keywords:** pancreatic adenocarcinoma, mutant KRAS and GATA6, chemotherapy

## Abstract

Background: Several pancreatic adenocarcinoma (PA) biomarkers beyond the traditional carbohydrate antigen (CA)19-9 have been identified but are lacking large-scale prospective validation. This prospective cohort study evaluated the prognostic impact of potential PA biomarkers. Methods: We enrolled 238 of 288 patients with histologically proven PA. We assessed candidate biomarkers, including CA19-9, germline *BRCA1/2*, and *ATM* mutations, as well as mutant *KRAS* circulating tumor DNA (ctDNA) in blood samples. Additionally, we evaluated the expression of SLC29A1 (hENT1), DCK, CES2, and GATA6. We examined the association of candidate biomarkers with progression-free survival (PFS) and overall survival (OS). Results: We analyzed biomarker efficacy in 200 (median age 65 years; 55% male) of the enrolled patients who received chemotherapy. A high mutant *KRAS* ctDNA concentration (hazard ratio [HR]: 1.508 and 95% confidence interval [CI]: 1.052–2.161 for PFS; HR: 1.796 and 95% CI: 1.203–2.681 for OS) and high CA19-9 level (HR: 1.647 and 95% CI: 1.177–2.306 for PFS; HR: 1.803 and 95% CI: 1.248–2.605 for OS) were associated with poor prognosis. High GATA6 RNA expression was linked to longer PFS (HR: 0.336 and 95% CI: 0.195–0.582) and OS (HR: 0.304 and 95% CI: 0.165–0.560). Conclusions: Plasma mutant *KRAS* ctDNA concentrations and GATA6 expression could serve as significant prognostic biomarkers in patients with PA, potentially guiding therapeutic decisions and prognostication.

## Simple Summary

Comprehensive studies on biomarkers in pancreatic adenocarcinoma (PA) remain scarce. The aim of our prospective cohort study was to assess the potential prognostic value of several biomarkers in real-world practice. Among several blood- and tissue-driven biomarkers, mutant *KRAS* and GATA6 RNA expression are significant prognostic biomarkers in PA. High mutant *KRAS* ctDNA concentration correlates with poor survival. Elevated GATA6 RNA expression is associated with longer survival outcomes. These biomarkers, along with CA19-9, can guide therapeutic decisions in PA.

## 1. Introduction

Pancreatic adenocarcinoma (PA) is the seventh leading cause of cancer-related deaths worldwide and has the poorest prognosis among cancers [1]. The overall 5-year survival rate is approximately 10% owing to its late diagnosis, with nearly 80% of patients presenting with locally advanced or metastatic disease [2]. Systemic chemotherapy is the mainstay of treatment for unresectable PA, which also plays an important role in resectable or borderline resectable PA as neoadjuvant or adjuvant therapy [3,4,5,6].

A significant challenge in improving PA treatment is the lack of precise predictive and prognostic biomarkers. Carbohydrate antigen 19-9 (CA19-9) is the most well-known serum biomarker for detecting PA and predicting chemotherapy response and survival [7,8]. However, this biomarker has limitations, including poor sensitivity, false positives in the presence of obstructive jaundice or other benign hepatic and pulmonary diseases, and false negatives in individuals with a Lewis-negative phenotype [9]. Therefore, there is a critical need for more reliable and specific biomarkers that can accurately predict prognosis and therapeutic response in PA patients.

Recent advances in molecular technology have enabled the discovery of novel biomarkers. Genetic alterations, such as the KRAS proto-oncogene, GTPase (*KRAS*) mutations (predominantly observed in PA), neuregulin 1 fusions, B-Raf proto-oncogene, serine/threonine kinase, and BRCA1 DNA repair-associated/BRCA2 DNA repair-associated (*BRCA1/2*) or ATM serine/threonine kinase (*ATM*) mutations, show promise for targeted therapies [10,11,12]. Additionally, microRNAs, circulating tumor DNA (ctDNA) and tumor cells, and epigenomic alterations may be potential biomarkers [13]. Among these, mutant *KRAS* (mKRAS) ctDNA has shown prognostic effects and is considered a highly promising biomarker [10].

Several proteins have also been evaluated as potential prognostic and predictive biomarkers. GATA binding protein 6 (GATA6), a transcription factor, plays a significant role in PA development and progression, and its expression level has a prognostic impact [14,15]. Human equilibrative nucleoside transporter 1 (hENT1) encoded by the SLC29A1, the primary gemcitabine transporter into cells, has been shown to predict the response to gemcitabine [16,17]. High levels of deoxycytidine kinase (DCK), which phosphorylates gemcitabine into its active form, are associated with improved survival in gemcitabine-treated patients [17]. Carboxylesterase 2 (CES2) and SMAD family member 4 have also been studied as protein biomarkers in PA [18,19].

Given the limitations of CA19-9 and the potential of these novel biomarkers, identifying reliable biomarkers that are adequately validated for real-world practice is warranted. This prospective cohort study aimed to evaluate the clinical significance of previously reported potential biomarkers in patients receiving chemotherapy for PA.

## 2. Methods

### 2.1. Patient Population

This study was approved by the institutional review board of the National Cancer Center, Republic of Korea (IRB number: NCC2019-0034) and was registered at ClinicalTrials.gov (identifier: NCT04281511). All participants provided written informed consent. Patients diagnosed with histologically proven pancreatic ductal adenocarcinoma between April 2019 and December 2020 were eligible for enrollment. We excluded patients with insufficient tissue for biomarker analysis, those who had been treated for another current malignancy, and those who did not provide informed consent to participate in the study. After enrollment, tissue and blood samples were collected to identify potential biomarkers before treatment. Tumor tissue samples were obtained from the surgical specimens of patients with resectable PA or the biopsy specimens of those with unresectable cancer before chemo- or radiotherapy. Primary resection specimens were used to evaluate tissue biomarkers in patients with recurrence after curative-intent surgery. Blood samples were collected to detect *KRAS*, *BRCA1/2*, and *ATM* mutations.

### 2.2. Treatment Strategy

The optimal treatment strategy was determined using a multidisciplinary team approach. After curative-intent surgery, patients who were fit underwent adjuvant chemotherapy with FOLFIRINOX (oxaliplatin, irinotecan, leucovorin, and 5-fluorouracil), gemcitabine plus capecitabine, or gemcitabine monotherapy, depending on their performance status. The neoadjuvant treatment consisted of chemotherapy with FOLFIRINOX and/or radiotherapy. Palliative chemotherapy was considered for patients with unresectable PA, and the regimen was determined at the discretion of the treating physician. Computed tomography and/or magnetic resonance imaging were performed every 8–12 weeks to evaluate treatment response or tumor recurrence. The tumors were evaluated using the Response Evaluation Criteria in Solid Tumors version 1.1.

### 2.3. Blood Biomarkers

Several blood biomarkers, including CA19-9, mKRAS ctDNA, and germline *BRCA1/2* and *ATM* mutations, were evaluated. Serum CA19-9 levels were measured using an immunoradiometric assay (Riakey CA19-9 kit; Shinjin Medics Inc., Goyang, Republic of Korea). The mKRAS ctDNA concentration was measured using a *KRAS* screening multiplex droplet digital polymerase chain reaction (ddPCR) kit (Bio-Rad, #1863506), which covers seven different *KRAS* mutations (G12A, G12C, G12D, G12R, G12S, G12V, and G13D) [11]. Detection of germline mutations in *BRCA1/2* and *ATM* was performed using a customized hereditary cancer panel (Celemics, Seoul, Republic of Korea) that targets coding sequences and intron–exon boundaries in more than 28 cancer predisposition genes, as previously reported [20].

### 2.4. Tissue Biomarkers

Several tissue biomarkers associated with prediction and prognosis in PA were evaluated. Hematoxylin and eosin-stained slides containing specimens from each tumor sample were reviewed, and representative tumor regions and corresponding formalin-fixed paraffin-embedded tissue blocks were selected for immunohistochemistry (IHC). IHC staining was performed using a BenchMark XT automated slide stainer (Ventana Medical Systems, Inc., Tucson, AZ, USA) for one to three paraffin-embedded blocks (median of two blocks) of each specimen. The primary antibodies used were rabbit polyclonal antibodies against human DCK (LS-B1825, LifeSpan Bioscience, Inc., Seattle, WA, USA), human hENT1 (11337-1-AP, ProteinTech Group, Inc., Chicago, IL, USA), human CES2 (HPA018897, Atlas Antibodies Inc., Stockholm, Sweden), and anti-GATA6 polyclonal antibody (HPA066629, Atlas Antibodies Inc.)

Representative images of the tissue biomarkers are shown in Supplementary Figure S1. Protein expression in tumor cells was scored by immunostaining intensity as follows: grade 0: not stained; grade 1: faintly positive; grade 2: weakly to moderately positive; and grade 3: strongly positive. IHC labeling was defined as positive when hENT1 and DCK were 1+ or higher, and CES2 was classified as low (negative, 1+) or high (2+, 3+). The expression of GATA6 was assessed using digital assistance as follows: GATA6 slides were scanned using an Aperio AT2 scanner (Leica Biosystems Inc., Vista, CA, USA) at 20× magnification, and open-source quantitative pathology and bioimage analysis software (QuPath v0.4.0; https://qupath.readthedocs.io) was used to view and analyze whole-slide images [21]. The proportion of GATA6-positive cells was determined by the ratio of positive cells to the total number of cells and divided into two groups: GATA6 high and low expression (Supplementary Figure S1).

*GATA6* RNA expression was adopted as a potential tissue biomarker because GATA6 expression was associated with the classical subtype, which had a better outcome and favorable response to 5-fluorouracil-based treatment [22]. Total RNA extractions were performed from FFPE samples using a Maxwell RSC RNA FFPE kit (Promega, Madison, WI, USA) on a Maxwell RSC 48 Instrument (Promega), according to the manufacturer’s protocol. The RNA was eluted in 40 µL of nuclease-free water. RNA concentrations, as well as the purity and contamination ratio values, were measured on a NanoDrop 8000 spectrophotometer (Thermo Scientific, Waltham, MA, USA), and 400 ng was used to synthesize cDNA using a cDNA EcoDry Premix kit (Takara Bio, Shiga, Japan) with random hexamer primer premix. The levels were assessed through polymerase chain reaction using the QX200^TM^ Droplet Digital^TM^ PCR System (Bio-Rad, Hercules, CA, USA). The mixture of the GATA6 FAM target probe and GUSB HEX reference probe, 2× ddPCR supermix (no dUTP), and cDNA template was adjusted to a final volume of 20.0 μL with diethylpyrocarbonate-treated water. Caco-2, U-2 OS, and HEK293T cells were used as positive controls. Relative *GATA6* RNA expression in each sample was normalized to GUSB using QuantaSoft Software version 1.6.6 (Bio-rad). Patients were divided into GATA6 high and low expression groups using the median value of gene expression (see the Supplementary Data File).

### 2.5. Statistical Analyses

This single-arm prospective observational study compared registry data, using overall survival (OS) as the primary endpoint. For biomarker-positive patients, an interim analysis was planned based on the O’Brien–Fleming alpha spending method, with a hazard ratio (HR) of 0.67, alpha assumed to be a two-sided type I error of 5%, and power assumed to be 80%. Considering a recruitment period of 36 months, a follow-up period of 24 months, and a 10% dropout rate, 119 patients were enrolled in the study. As 50% of the patients with PA were expected to have at least one positive biomarker, 238 study participants were enrolled in the study.

OS was defined as the time from patient enrollment to death and progression-free survival (PFS) as the time from patient enrollment until tumor progression or death. For patients who underwent curative-intent surgery, recurrence-free survival (RFS) was defined as the time from surgical resection to the onset of tumor recurrence or death. Survival curves were estimated using the Kaplan–Meier method and compared using log-rank tests. To identify biomarkers with prognostic significance, a Cox proportional hazards model was used, adjusting for clinical variables with prognostic significance at baseline. CA19-9 levels were dichotomized with a known cutoff concentration of 37 U/mL, and mKRAS ctDNA concentration was dichotomized with a previously described cutoff value of 160 copies/mL [11]. All analyses were performed using SAS version 9.4 (SAS Institute Inc., Cary, NC, USA) and R version 4.3.3 (R Foundation for Statistical Computing, Vienna, Austria).

## 3. Results

### 3.1. Patient Characteristics

**Figure 1:**
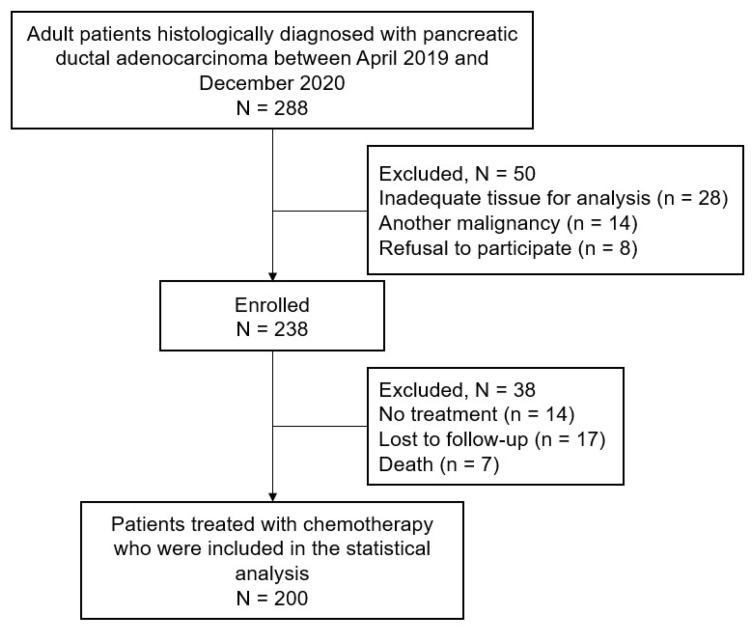
Participant enrolment flow diagram. Adult patients with a histologic diagnosis of pancreatic ductal adenocarcinoma were screened, and those who received chemotherapy were included in the final analysis.

Of the 288 patients with histologically confirmed pancreatic ductal adenocarcinoma who were screened, 238 were enrolled. Of these, 200 patients who received at least one cycle of chemotherapy were included in the final analysis (Figure 1). The baseline characteristics are shown in Table 1. The median age was 65 years (range: 45–90 years) and 110 patients (55%) were male. Most patients had an Eastern Cooperative Oncology Group performance status score of 0. At enrollment, 46 (23%) had resectable PA, 58 (29%) had borderline resectable or locally advanced PA, and 96 (48%) had metastatic PA. Most patients received palliative chemotherapy, with FOLFIRINOX being the most common regimen.

### 3.2. Baseline Distribution of Candidate Biomarkers

The distribution of candidate biomarkers is summarized in Supplementary Table S1. The mKRAS ctDNA concentration was significantly higher in patients with metastatic disease than those with localized disease (resectable, borderline resectable, and locally advanced) (Supplementary Figure S2A). Germline mutations in *BRCA1/2* were observed in 22 patients (3 pathogenic variants and 19 of uncertain significance) and *ATM* mutations in 15 patients (4 pathogenic variants and 11 of uncertain significance). Serum CA19-9 levels were elevated in 65% of patients.

Among the tissue biomarkers, GATA6 expression was elevated in 53 patients at the RNA level and in 77 patients according to IHC measurements (Supplementary Table S1). GATA6 RNA and tissue (IHC) expression levels were correlated with each other and associated with the cancer stage (Supplementary Figure S2B–D). Positivity for hENT1 and DCK was observed in 88 (44%) and 80 (40%) patients, respectively. High CES2 expression was observed in 32 (16%) patients (Supplementary Table S1).

### 3.3. Prognostic Efficacy of Candidate Biomarkers

The median duration of follow-up was 35.5 months (range: 3.2–47.8 months). In total, 145 (72.5%) patients died, and 169 (84.5%) experienced disease progression. The median OS was 9.3 months (95% confidence interval [CI]: 7.7–11.9) for metastatic disease and 27.7 months (95% CI: 21.4–not applicable) for localized disease. The median PFS was 5.7 months (95% CI: 4.7–6.6) for metastatic disease and 13.9 months (95% CI: 12.1–21.4) for localized disease (Supplementary Figure S3).

**Figure 2:**
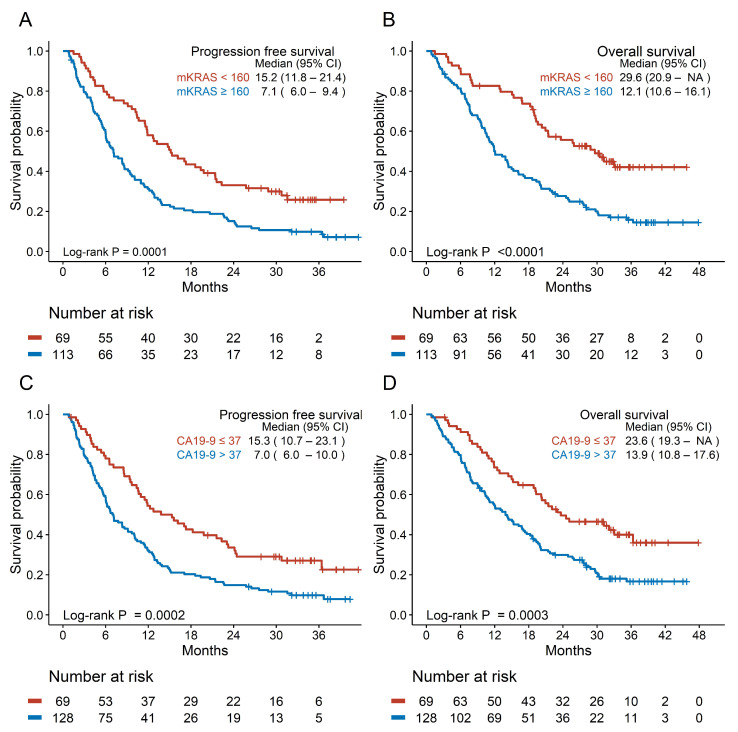
Survival analysis according to the blood biomarkers. (**A**) Progression-free survival and (**B**) overall survival according to mutant *KRAS* status. (**C**) Progression-free survival and (**D**) overall survival according to CA19-9 level. CA19-9: carbohydrate antigen 19-9; CI: confidence interval; *KRAS*: KRAS proto-oncogene, GTPase; mKRAS: mutant *KRAS*; NA: not assessed.

Patients with mKRAS ctDNA concentrations >160 copies/mL had significantly shorter PFS (7.1 vs. 15.2 months) and OS (12.1 vs. 29.6 months) than those with concentrations <160 copies/mL (Figure 2A,B). Patients with elevated CA19-9 levels had shorter PFS (7.0 vs. 15.3 months, respectively) and OS (13.9 vs. 23.6 months, respectively) than those with normal levels (Figure 2C,D). Patients with pathogenic germline variants showed a trend toward longer PFS and OS, although this was not statistically significant (Supplementary Figure S4).

**Figure 3:**
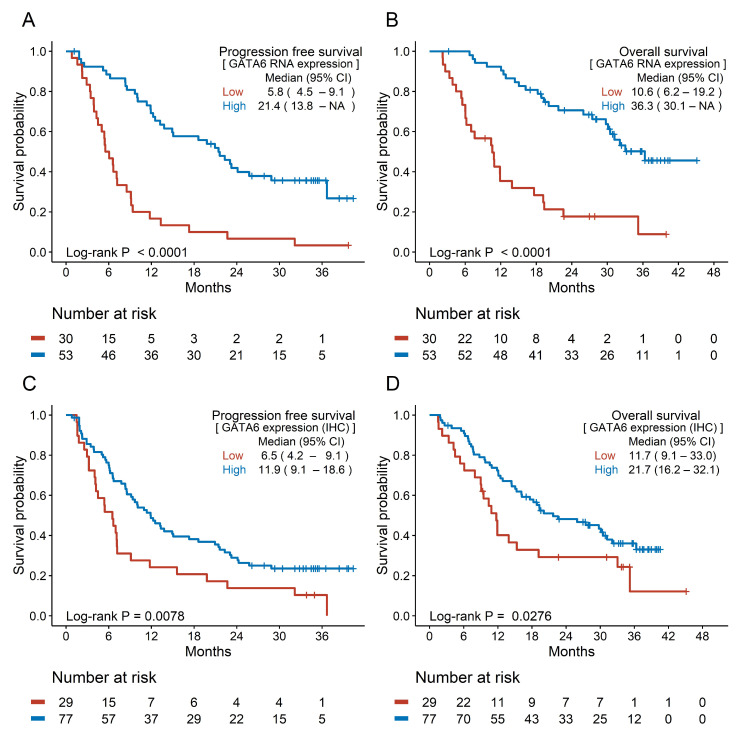
Survival analysis according to the tissue biomarkers. (**A**) Progression-free survival and (**B**) overall survival according to GATA6 RNA expression. (**C**) Progression-free survival and (**D**) overall survival according to GATA6 tissue expression (IHC). CI: confidence interval; GATA6: GATA binding protein 6; IHC: immunohistochemistry; NA: not assessed.

Among tissue biomarkers, patients with high GATA6 expression showed better survival outcomes than those with low GATA6 expression. The median PFS and OS in patients with high *GATA6* RNA expression were 21.4 months and 36.3 months compared with 5.8 months and 10.6 months in those with low RNA expression, respectively (Figure 3A,B). High GATA6 expression was associated with longer survival (median PFS: 11.9 vs. 6.5 months; median OS: 21.7 vs. 11.7 months) (Figure 3C,D). No significant association was found between hENT1 and CES2 expression and survival outcomes, whereas DCK positivity was associated with longer OS (20.1 vs. 12.0 months) (Supplementary Figure S5).

### 3.4. Cox Regression Analysis of Survival

Cancer stage was significantly associated with both PFS and OS among clinical factors (Supplementary Table S2). Among biomarkers, mKRAS ctDNA concentration, CA19-9 level, and *GATA6* RNA expression were significantly associated with PFS in the univariable Cox model, and DCK was significantly associated with OS (Table 2). After adjusting for cancer stage, high mKRAS ctDNA concentration (HR: 1.508 and 95% CI: 1.052–2.161 for PFS; HR: 1.796 and 95% CI: 1.203–2.681 for OS) and CA19-9 level (HR: 1.647 and 95% CI: 1.177–2.306 for PFS; HR: 1.803 and 95% CI: 1.248–2.605 for OS) remained significantly associated with poor survival. High *GATA6* RNA expression was associated with better survival (HR: 0.336 and 95% CI: 0.195–0.582 for PFS; HR: 0.304 and 95% CI: 0.165–0.560 for OS). DCK expression was not significantly associated with OS after adjusting for cancer stage.

### 3.5. Subgroup Analysis Based on the Treatment Setting

We further evaluated the prognostic value of mKRAS ctDNA and GATA6 according to treatment modality owing to the heterogeneity of cancer stages among patients. In patients who underwent curative-intent surgery before/after chemotherapy, a high mKRAS ctDNA concentration was associated with shorter RFS (14.3 vs. 25.5 months, *p* = 0.068) (Supplementary Figure S6A). Conversely, high *GATA6* RNA expression indicated a trend toward longer RFS in patients with resected PA (23.9 vs. 13.2 months, *p* = 0.056) (Supplementary Figure S6B). Among clinical factors, the pathological T and N stages were significant prognostic factors; however, they were not significantly associated with the *KRAS* level or *GATA6* RNA expression (Supplementary Table S3 and Supplementary Figure S7). In patients who received palliative chemotherapy, a high mKRAS ctDNA concentration was associated with poor survival outcomes (6.0 vs. 10.4 months for PFS; 10.6 vs. 19.6 months for OS) (Supplementary Figure S6C,D).

## 4. Discussion

Our findings demonstrate that among several candidate biomarkers, mKRAS ctDNA concentration and *GATA6* RNA expression were meaningful prognostic markers in PA, alongside the CA19-9 level. A high mKRAS ctDNA concentration and elevated CA19-9 level were associated with poor PFS and OS, whereas high *GATA6* RNA expression correlated with better survival outcomes. In resected PA, these markers were associated with RFS after surgery, and in palliative chemotherapy, a high mKRAS ctDNA concentration was significantly associated with poor OS.

ctDNA is increasingly recognized as a promising cancer biomarker for diagnosis, treatment, and prognosis because it can identify tumor-specific abnormalities. *KRAS* mutations are key oncogenic drivers of PA and are found in almost all patients with this disease. mKRAS ctDNA showed significant associations with survival in a series of studies and meta-analyses [23,24]. Guven et al. demonstrated that positive ctDNA was associated with lower RFS/PFS and OS in both localized and advanced PA in a pooled analysis, and the *KRAS* mutation was a target for ctDNA analyses [23].

Although most previous studies focused on the detection of mKRAS ctDNA, several have used ctDNA as a continuous biomarker with mutant allele fraction (MAF) values [8,25,26]. Quantification of the biomarker may help generalize the clinical use of mKRAS ctDNA for real-world applications, similar to serum CA19-9. Our previous research indicated that a high mKRAS ctDNA concentration was associated with shorter PFS and OS, and this study confirms its prognostic value and clinical applicability, aligning with other studies [11]. Further study into the prognostic value of the mKRAS subtype may help inform treatment strategies given the recent advances in KRAS inhibitors [27].

Regarding GATA6 expression, our findings align with previous research, highlighting its role as a favorable prognostic marker. Duan et al. demonstrated that high GATA6 IHC values are associated with improved clinical outcomes in advanced PA [28]. GATA6 IHC values are correlated with *GATA6* RNA gene expression, which is consistent with our results. O’Kane et al. showed that GATA6 expression, measured using both RNA sequencing and in situ hybridization, was a prognostic factor in multivariate analysis [29]. Our results further support the prognostic value of GATA6 by showing significant associations between high *GATA6* RNA expression and longer PFS and OS, reinforcing its potential as a prognostic biomarker for PA.

The strengths of our study include the comprehensive assessment of potential biomarkers and its prospective cohort design, which allowed for a rigorous evaluation of the prognostic impact. Among the candidate biomarkers, mKRAS ctDNA and GATA6 were identified as significant prognostic factors, along with CA19-9, consistent with previous studies, providing a strong rationale for their clinical implications. The use of advanced molecular techniques, such as ddPCR for qualitative mKRAS detection and digital quantification for GATA6 assessment, further enhanced the robustness and reliability of our findings.

However, this study has some limitations. First, although we performed a comprehensive assessment of multiple biomarkers in a large number of patients, some tissue biomarkers could not be assessed simultaneously owing to limited tissue volume. Second, for germline *BRCA1/2* or *ATM* mutations, significance was not achieved owing to their low prevalence. Germline mutation carriers of homologous recombination repair genes, such as *ATM*, *BRCA1/2*, and *PALB2,* are considered predictive and prognostic biomarkers, especially in patients receiving platinum-based chemotherapy [30,31]. Finally, although our study confirms the clinical significance of the biomarkers, it does not elucidate a causal relationship based on biological mechanisms. *KRAS* is a representative oncogenic gene driver of PA, whereas GATA6, a transcription factor for pancreatic development, correlates with the molecular subtypes of PA and plays an inhibitory role in epithelial–mesenchymal transition, which may explain the opposite trends in PA prognosis [22,29].

## 5. Conclusions

Our findings highlight the significant prognostic value of mKRAS ctDNA and *GATA6* RNA expression in patients with PA undergoing chemotherapy. These biomarkers provide valuable insights into patient prognosis and guide therapeutic decision-making, ultimately improving personalized treatment strategies and outcomes in patients with PA.

## Figures and Tables

**Table 1:** Baseline patient characteristics.

Variable		N = 200
Age (years)	Median (range)	65 (45–90)
Sex	Female	90 (45.0%)
	Male	110 (55.0%)
ECOG-PS	0	128 (64.0%)
	1 or more	72 (36.0%)
Tumor location	Head or neck or uncinate process	92 (46.0%)
	body or tail	108 (54.0%)
Stage	Resectable	46 (23.0%)
	Borderline resectable/locally advanced	58 (29.0%)
	Metastatic/recurrent	96 (48.0%)
Surgery	No	125 (62.5%)
	Yes	75 (37.5%)
Chemotherapy	Neoadjuvant	21 (10.5%)
	Adjuvant	45 (22.5%)
	Palliative	134 (67.0%)
Regimen	FOLFIRINOX	131 (65.5%)
	Gemcitabine + nab-paclitaxel	40 (20.0%)
	Other 5-fluorouracil-based	12 (6.0%)
	Other gemcitabine-based	17 (8.5%)

ECOG-PS: European Cooperative Oncology Group Performance Status; FOLFIRINOX: oxaliplatin, irinotecan, leucovorin, and 5-fluorouracil.

**Table 2:** Cox proportional hazards model showing associations between biomarkers and survival.

Variable		N	Progression-Free Survival					Overall Survival						
			Event	Univariable		Adjusted for Stage		Event	Univariable		Adjusted for Stage		Adjusted for Sex and Stage	
				HR (95% CI)	*p*-Value	HR (95% CI)	*p*-Value		HR (95% CI)	*p*-Value	HR (95% CI)	*p*-Value	HR (95% CI)	*p*-Value
CA19-9 level (U/mL)	≤37	69	50	1 (ref)		1 (ref)		40	1 (ref)		1 (ref)		1 (ref)	
	>37	128	116	1.866 (1.336–2.605)	<0.001	1.647 (1.177–2.306)	0.0036	103	1.942 (1.345–2.802)	<0.001	1.803 (1.248–2.605)	0.0017	1.764 (1.22–2.551)	0.0026
mKRAS ctDNA concentration	<160	69	50	1 (ref)		1 (ref)		37	1 (ref)		1 (ref)		1 (ref)	
(copies/mL)	≥160	113	103	1.965 (1.398–2.763)	<0.001	1.508 (1.052–2.161)	0.0253	94	2.344 (1.598–3.437)	<0.001	1.796 (1.203–2.681)	0.0042	1.813 (1.215–2.704)	0.0036
*BRCA* mutation	Not detected	175	151	1 (ref)		1 (ref)		131	1 (ref)		1 (ref)		1 (ref)	
	VUS/PV	22	15	0.717 (0.421–1.219)	0.219	0.724 (0.424–1.235)	0.235	12	0.650 (0.360–1.175)	0.153	0.640 (0.354–1.158)	0.140	0.694 (0.382–1.261)	0.230
*ATM* mutation	ND	182	155	1 (ref)		1 (ref)		133	1 (ref)		1 (ref)		1 (ref)	
	VUS/PV	15	11	0.707 (0.384–1.305)	0.267	0.739 (0.401–1.365)	0.334	10	0.778 (0.409–1.479)	0.443	0.796 (0.418–1.516)	0.487	0.823 (0.432–1.568)	0.553
hENT1	Negative	40	33	1 (ref)		1 (ref)		31	1 (ref)		1 (ref)		1 (ref)	
	Positive	88	72	0.905 (0.598–1.368)	0.6354	0.904 (0.598–1.367)	0.6321	60	0.748 (0.484–1.156)	0.1909	0.682 (0.440–1.055)	0.0853	0.706 (0.455–1.093)	0.1184
DCK	Negative	48	43	1 (ref)		1 (ref)		41	1 (ref)		1 (ref)		1 (ref)	
	Positive	80	62	0.724 (0.490–1.070)	0.1056	1.045 (0.692–1.579)	0.8327	50	0.575 (0.380–0.870)	0.0088	0.788 (0.513–1.210)	0.2767	0.788 (0.513–1.21)	0.2767
CES2	Low	93	76	1 (ref)		1 (ref)		64	1 (ref)		1 (ref)		1 (ref)	
	High	32	23	0.772 (0.484–1.231)	0.2774	0.850 (0.532–1.358)	0.4966	19	0.769 (0.461–1.284)	0.3154	0.838 (0.501–1.400)	0.4990	0.978 (0.579–1.652)	0.9328
GATA6 (RNA)	Low	30	29	1 (ref)		1 (ref)		25	1 (ref)		1 (ref)		1 (ref)	
	High	53	34	0.266 (0.159–0.447)	<0.0001	0.336 (0.195–0.582)	<0.0001	24	0.225 (0.126–0.402)	<0.0001	0.304 (0.165–0.560)	0.0001	0.269 (0.144–0.502)	<0.0001
GATA6 (Tissue)	Low	29	27	1 (ref)		1 (ref)		22	1 (ref)		1 (ref)		1 (ref)	
	High	77	58	0.541 (0.341–0.857)	0.0088	0.968 (0.587–1.596)	0.8970	47	0.568 (0.341–0.945)	0.0294	1.301 (0.749–2.260)	0.3507	1.302 (0.755–2.243)	0.3423

ATM: ATM serine/threonine kinase; BRCA: breast cancer gene; CA19-9: carbohydrate antigen 19-9; CES2: carboxylesterase 2; CI: confidence interval; DCK: deoxycytidine kinase; GATA6: GATA binding protein 6; hENT1: human equilibrative nucleoside transporter 1; HR: hazard ratio; IHC: immunohistochemistry; KRAS: KRAS proto-oncogene, GTPase; PV: pathogenic variant; VUS: variants of unknown significance.

## Data Availability

All data used and analyzed during the current study are available from the corresponding author upon reasonable request.

## Supplementary Materials

The following supporting information can be downloaded at: https://www.mdpi.com/article/10.3390/cancers17050896/s1, Figure S1: Representative images of the tissue biomarkers; Figure S2: Distribution of mutant KRAS status and GATA6 expression; Figure S3: Survival analysis according to cancer stage; Figure S4: Survival analysis according to the blood biomarkers. Figure S5: Survival analysis according to the tissue biomarkers. Figure S6: Analysis of subgroups according to treatment type; Figure S7: Distribution of mutant KRAS status and GATA6 RNA expression according to pathological T and N stage in surgical patients; Table S1: Biomarker distributions; Table S2: Cox hazard model of baseline characteristics; Table S3: Cox Hazard Model of Baseline Characteristics in Surgical Patients.

## Abbreviations

PA: pancreatic adenocarcinoma; CA19-9: carbohydrate antigen 19-9; *KRAS*: KRAS proto-oncogene, GTPase; ctDNA: circulating tumor DNA; mKRAS: mutant *KRAS*; *BRCA1/2*: BRCA1 DNA repair-associated and BRCA2 DNA repair-associated; *ATM*: ATM serine/threonine kinase; GATA6: GATA binding protein 6; SLC29A1: solute carrier family 29 member 1; hENT1: human equilibrative nucleoside transporter 1; DCK: deoxycytidine kinase; ddPCR: droplet digital polymerase chain reaction; IHC: immunohistochemistry; OS: overall survival; PFS: progression-free survival; RFS: recurrence-free survival; HR: hazard ratio; CI: confidence interval.
