# Supplementary material for: Mutant KRAS and GATA6 Stratify Survival in Patients Treated with Chemotherapy for Pancreatic Adenocarcinoma: A Prospective Cohort Study
Source: Cancers (Basel). 2025 Mar 5;17(5):896. doi: 10.3390/cancers17050896 (PMC11899085; doi:10.3390/cancers17050896)
Supplement: Supplementary file 1 [file cancers-17-00896-s001.zip › cancers-3490091-supplementary.pdf]

cancers MDPI

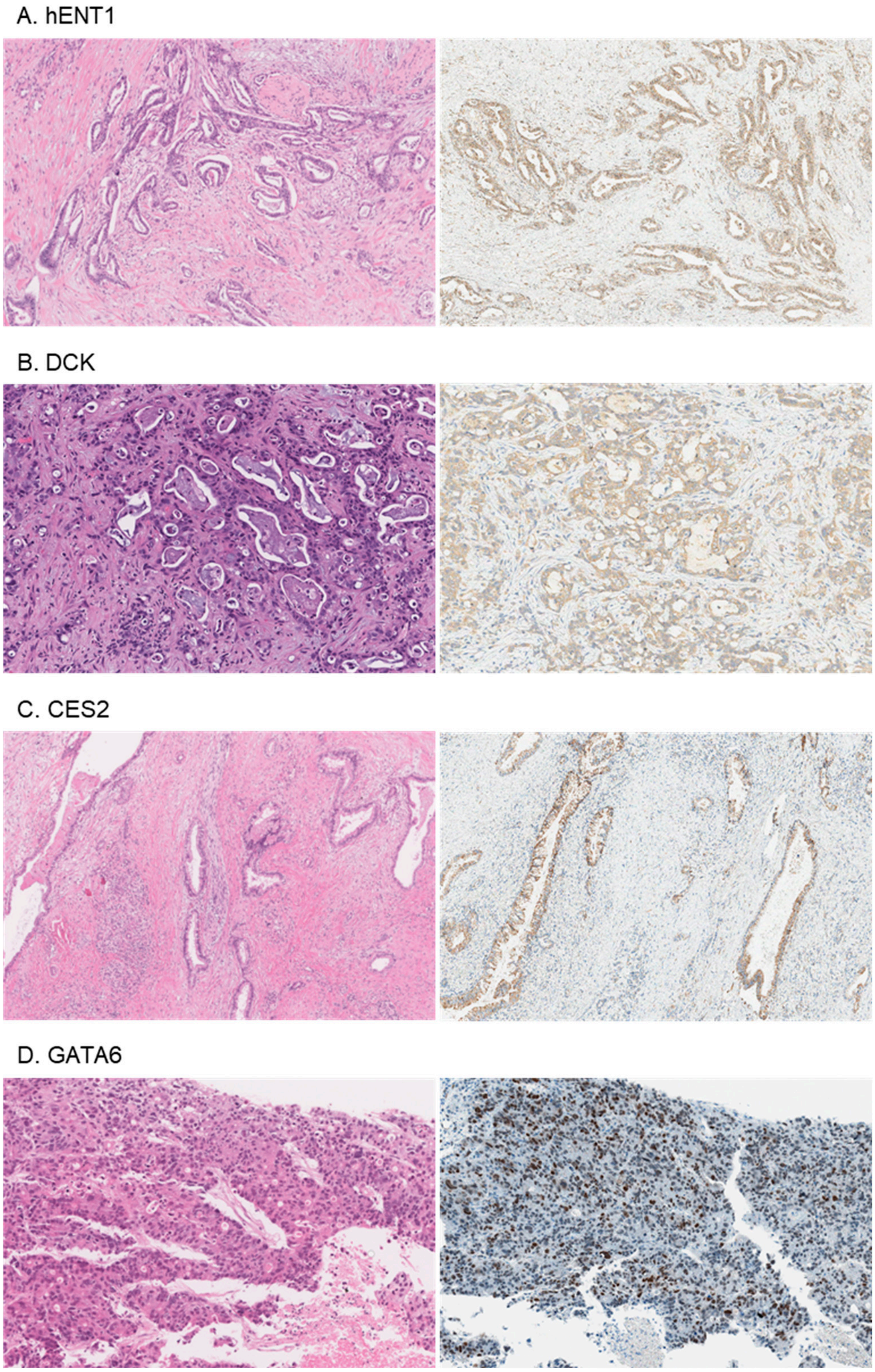

Figure S1. Presentative images of tissue biomarkers.

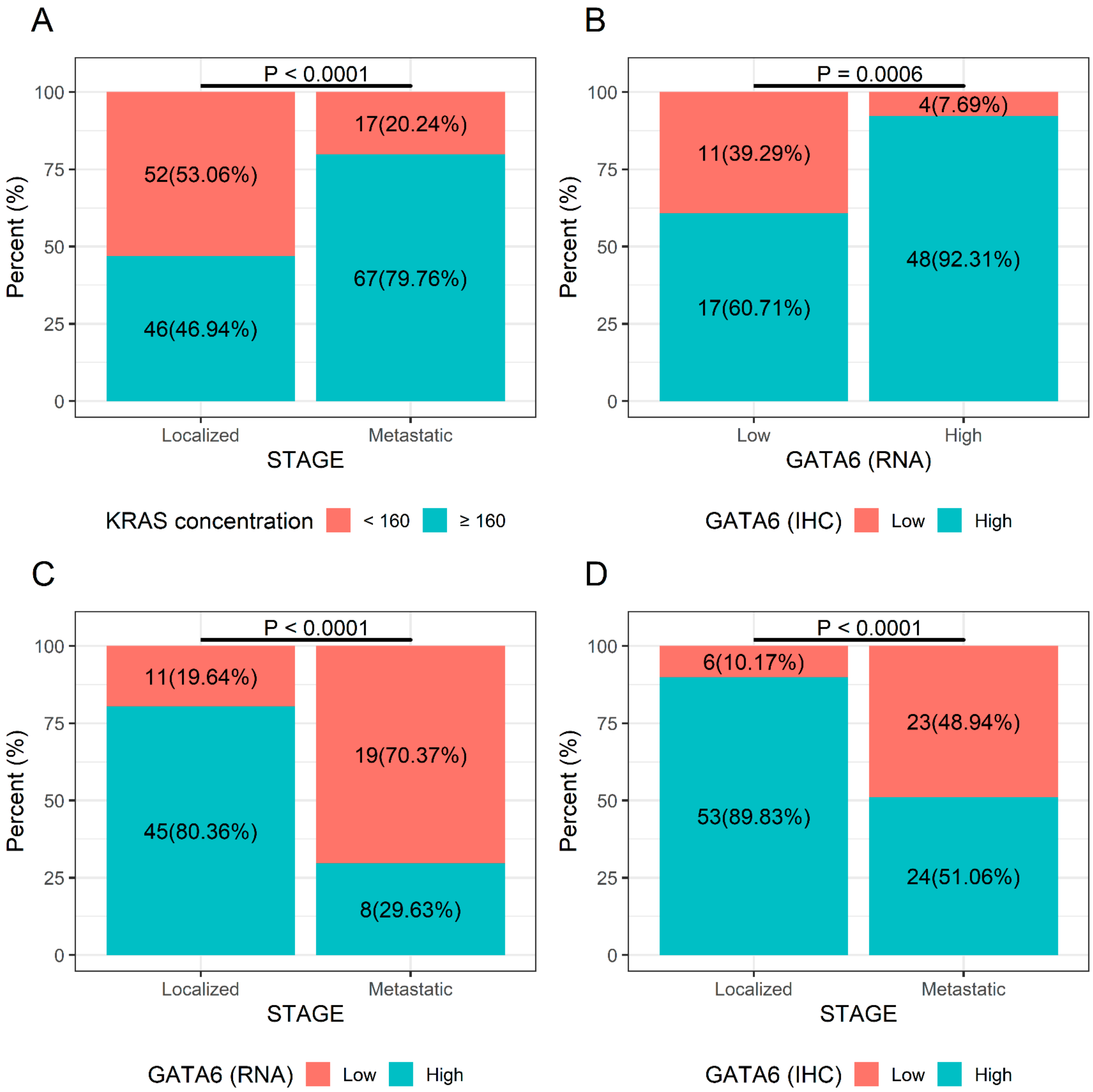

Figure S2. Distribution of mutant KRAS status and GATA6 expression. (A) Mutant KRAS according to cancer stage. (B) Correlation between GATA6 RNA and IHC expression. (C) GATA6 RNA expression and (D) GATA6 IHC expression according to cancer stage

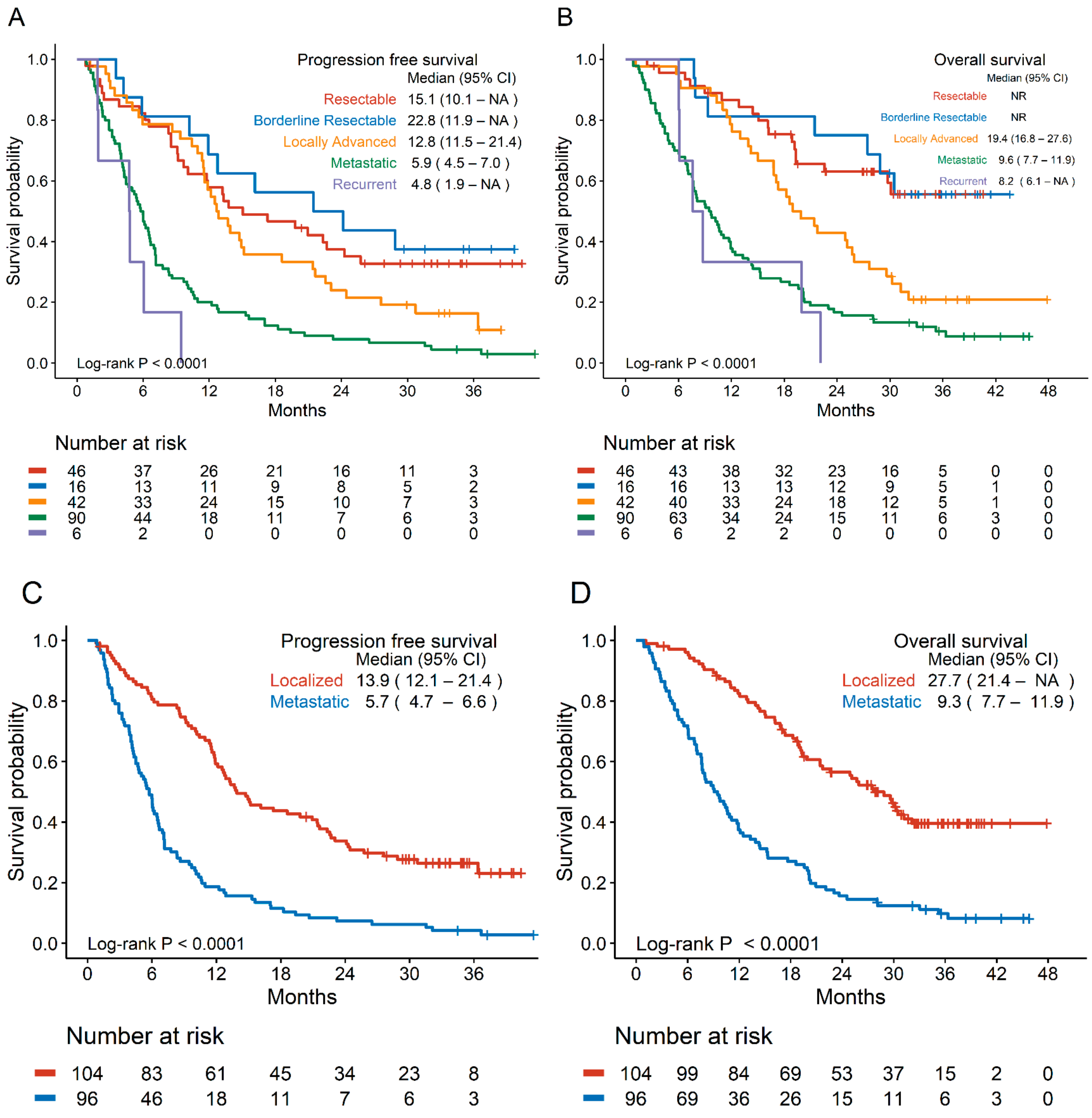

Figure S3. Survival analysis According to Cancer Stage. (A) Progression-free survival and (B) overall survival according to cancer stages. (C) Progression-free survival and (D) overall survival according to disease group (localized or metastatic).

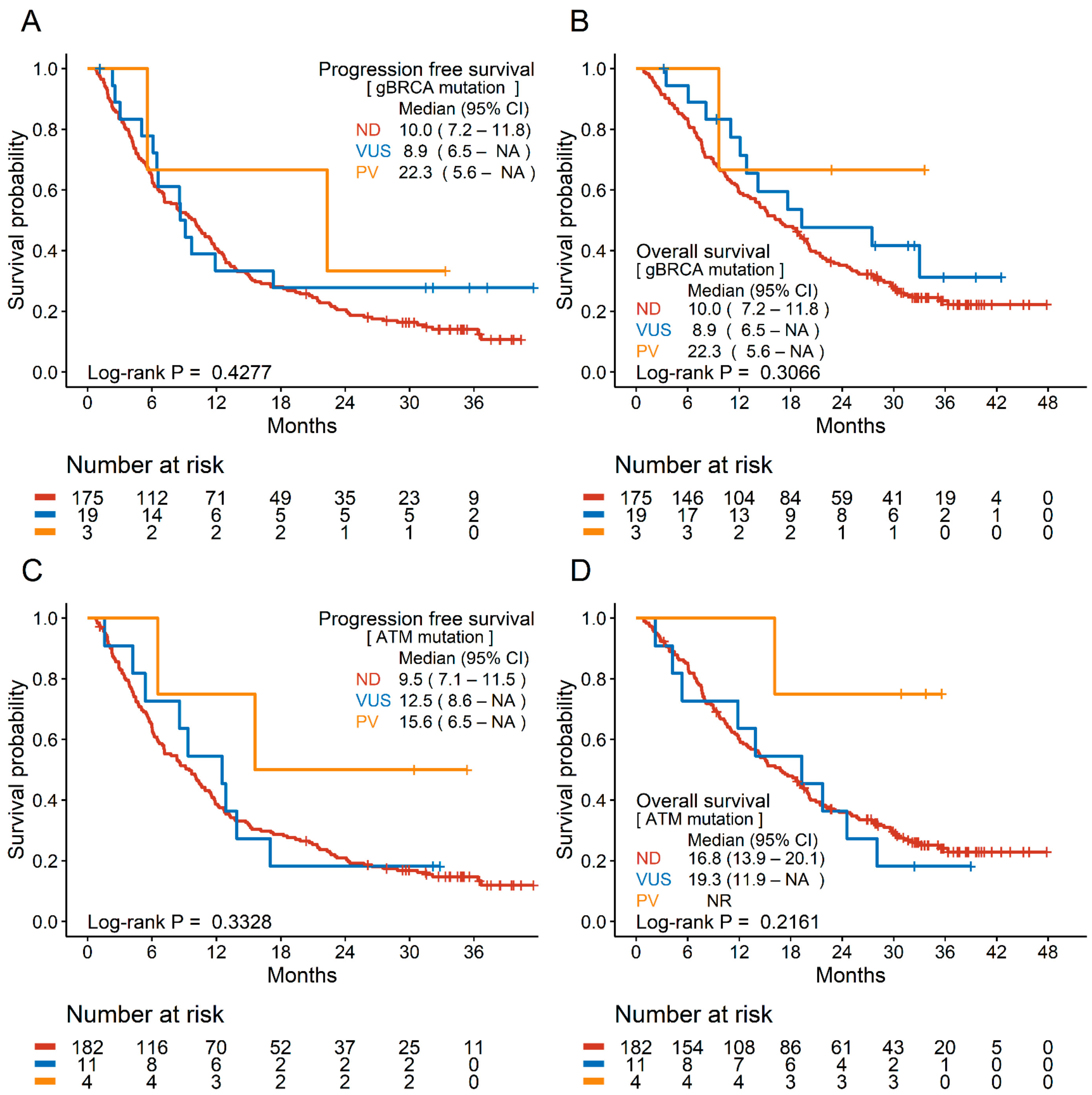

Figure S4. Survival analysis based on the blood biomarkers. (A) Progression-free survival and (B) overall survival according to germline BRCA mutation. (C) Progression-free survival and (D) overall survival according to germline ATM mutation.

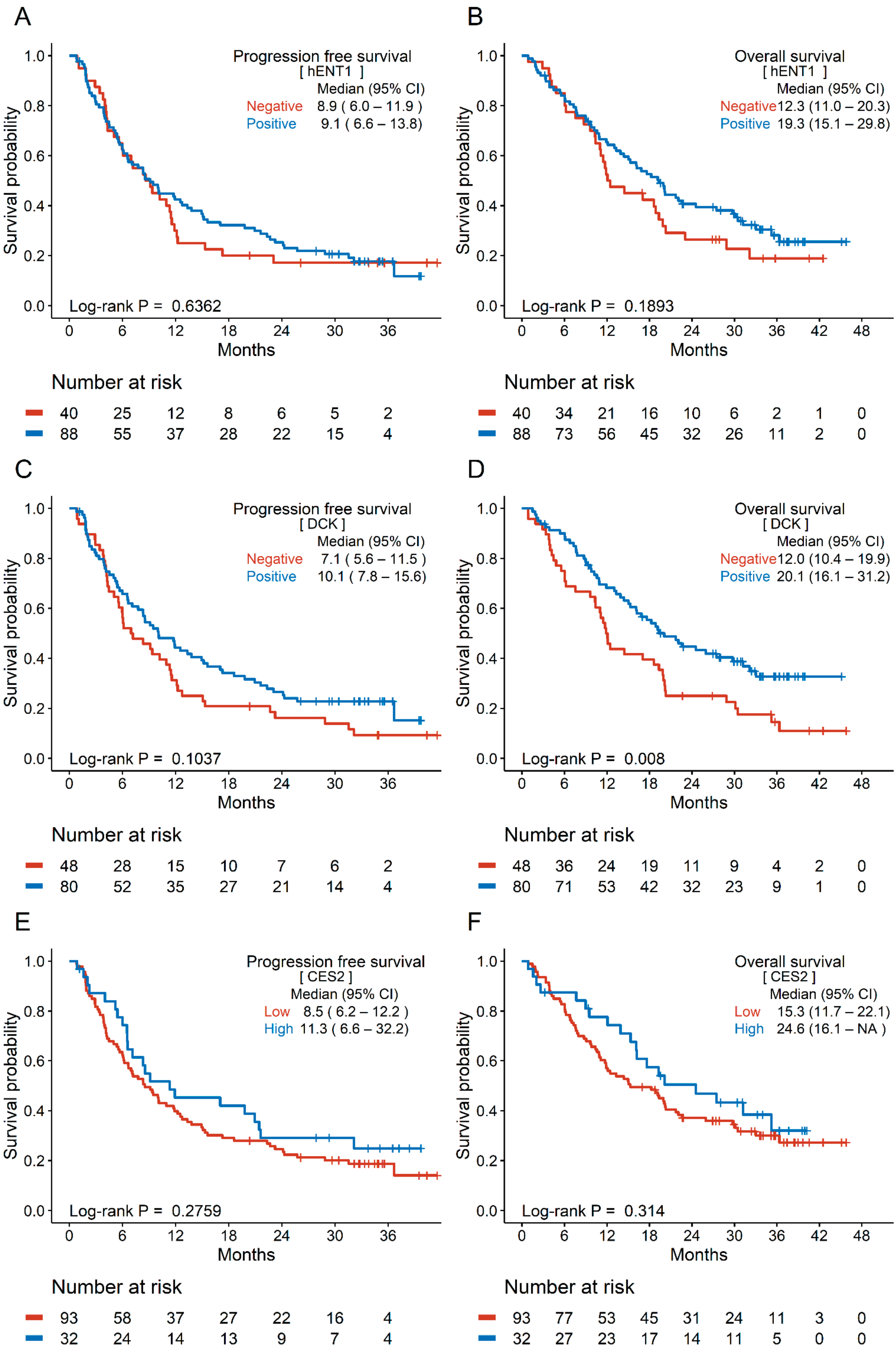
A
Progression free survival
[hENT1]
Median (95% CI)
Negative 8.9 (6.0 – 11.9)
Positive 9.1 (6.6 – 13.8)
Log-rank P = 0.6362
Survival probability
Months
Number at risk
40 25 12 8 6 5 2
88 55 37 28 22 15 4
B
Overall survival
[hENT1]
Median (95% CI)
Negative 12.3 (11.0 – 20.3)
Positive 19.3 (15.1 – 29.8)
Log-rank P = 0.1893
Survival probability
Months
Number at risk
40 34 21 16 10 6 2 1 0
88 73 56 45 32 26 11 2 0
C
Progression free survival
[DCK]
Median (95% CI)
Negative 7.1 (5.6 – 11.5)
Positive 10.1 (7.8 – 15.6)
Log-rank P = 0.1037
Survival probability
Months
Number at risk
48 28 15 10 7 6 2
80 52 35 27 21 14 4
D
Overall survival
[DCK]
Median (95% CI)
Negative 12.0 (10.4 – 19.9)
Positive 20.1 (16.1 – 31.2)
Log-rank P = 0.008
Survival probability
Months
Number at risk
48 36 24 19 11 9 4 2 0
80 71 53 42 32 23 9 1 0
E
Progression free survival
[CES2]
Median (95% CI)
Low 8.5 (6.2 – 12.2)
High 11.3 (6.6 – 32.2)
Log-rank P = 0.2759
Survival probability
Months
Number at risk
93 58 37 27 22 16 4
32 24 14 13 9 7 4
F
Overall survival
[CES2]
Median (95% CI)
Low 15.3 (11.7 – 22.1)
High 24.6 (16.1 – NA)
Log-rank P = 0.314
Survival probability
Months
Number at risk
93 77 53 45 31 24 11 3 0
32 27 23 17 14 11 5 0 0

Figure S5. Survival analysis based on the tissue biomarkers. Progression-free survival and overall survival according to (A-B) hENT1 (C-D) DCK (E-F) CES2.

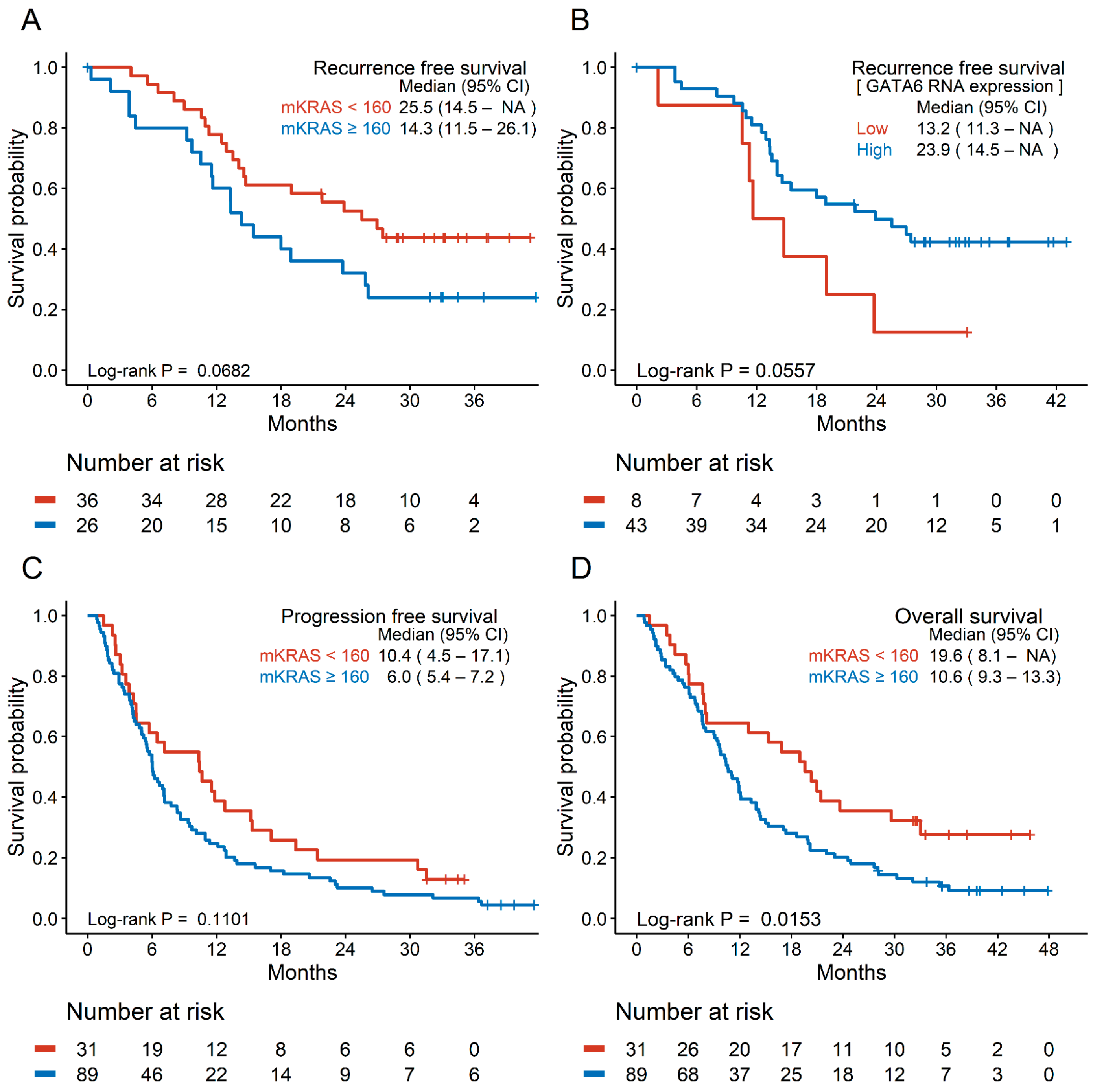

Figure S6. Analysis of subgroups according to type of treatment. Recurrent-free survival according to (A) mutant KRAS ctDNA concentration and (B) GATA6 RNA expression in patients underwent surgery. (C) Progression-free survival and (D) overall survival according to mutant KRAS ctDNA in patients who received palliative chemotherapy.

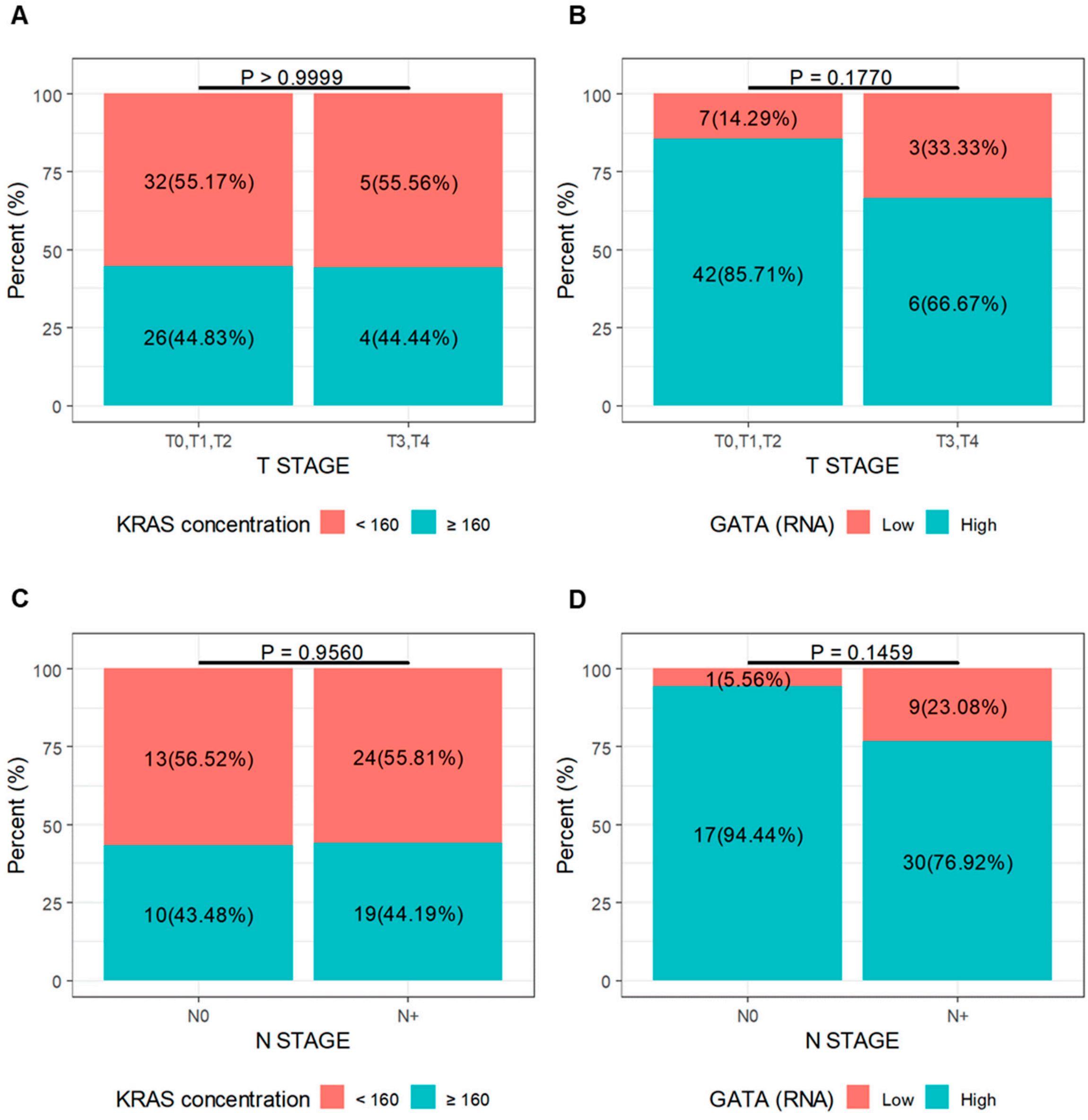

Figure S7. Distribution of mutant KRAS status and GATA6 RNA expression according to pathological T and N stage in surgical patients. Correlation between (A) mutant KRAS concentration or (B) GATA6 expression according to T stage. Correlation between (C) mutant KRAS concentration or (D) GATA6 expression according to N stage.

Table S1. Biomarker Distributions.

Variable		N = 200
***mKRAS* concentration (copies/mL)**	<160	69 (37.9%)
	≥160	113 (62.1%)
***BRCA1*/2 mutation**	PV/VUS	3/19 (11.0%)
	Not detected	175 (87.5%)
***ATM* mutation**	PV/VUS	4/11 (7.5%)
	Not detected	182 (91.0%)
**CA19-9** (U/mL)	≤37	69 (35.0%)
	>37	128 (65.0%)
**GATA6 RNA expression**	Low	30 (15.0%)
	High	53 (26.5%)
	Not applicable	115 (57.5%)
**GATA6 expression (IHC)**	Low	29 (14.5%)
	High	77 (38.5%)
	Not applicable	94 (47.0%)
**hENT1**	Negative	40 (20.0%)
	Positive	88 (44.0%)
	Not applicable	72 (36.0%)
**DCK**	Negative	48 (24.0%)
	Positive	80 (40.0%)
	Not applicable	72 (36.0%)
**CES2**	Low	93 (46.5%)
	High	32 (16.0%)
	Not applicable	75 (37.5%)

ATM, ATM serine/threonine kinase; BRCA, BReast CAncer gene; CA19-9, carbohydrate antigen 19-9; CES2, carboxylesterase 2; DCK, deoxycytidine kinase; GATA6, GATA binding protein 6; hENT1, human equilibrative nucleoside transporter 1; IHC, immunohistochemistry; mKRAS, mutant Kirsten rat sarcoma viral oncogene homologue; PV, pathogenic variant; VUS, variants of unknown significance

Table S2. Cox Hazard Model of Baseline Characteristics.

Variable		N	Progression-free survival			Overall survival				
			Event	Univariable		Event	Univariable		Multivariable	
				HR (95%CI)	p-value		HR (95%CI)	p-value	HR (95%CI)	p-value
**Stage**	**Localized**	**104**	**76**	1(ref)		58	1(ref)		1(ref)	
	Metastatic	96	93	2.807 (2.054–3.835)	<0.001	87	3.089 (2.205–4.327)	<0.001	3.160 (2.253–4.431)	<0.001
**Sex**	Female	90	72	1(ref)		60	1(ref)		1(ref)	
	Male	110	97	1.225 (0.902–1.664)	0.193	85	1.473 (1.058–2.052)	0.022	1.552 (1.113–2.165)	0.0096
**Age**		200	169	1.004 (0.987–1.021)	0.636	145	1.008 (0.990–1.026)	0.396		
**Tumor location**	Head	92	79	1(ref)		65	1(ref)			
	Body or tail	108	90	1.011 (0.747–1.369)	0.941	80	1.162 (0.837–1.611)	0.370		
**ECOG-PS**	0	128	105	1(ref)		89	1(ref)			
	1 or more	72	64	1.168 (0.855–1.596)	0.327	56	1.197 (0.857–1.673)	0.291		

CI, confidence interval; ECOG-PS, European Cooperative Oncology Group Performance Status; HR, hazard ratio.

Table S3. Cox Hazard Model of Baseline Characteristics in Surgical Patients.

Variable		N	Progression-free survival			Overall survival		
			Event	Univariable		Event	Univariable	
				HR (95%CI)	p-value		HR (95%CI)	p-value
**Stage**	Localized	69	45	1(ref)		30	1(ref)	
	Metastatic	4	3	1.853 (0.574-5.982)	0.3025	3	2.391 (0.837-6.83)	0.1035
**Sex**	Female	34	20	1(ref)		13	1(ref)	
	Male	39	28	1.182 (0.663-2.109)	0.5708	20	1.461 (0.742-2.878)	0.2732
**Age**		73	48	1.01 (0.979-1.041)	0.5387	33	1.025 (0.988-1.064)	0.1942
**Tumor location**	Head	36	26	1(ref)		17	1(ref)	
	Body or tail	37	22	0.72 (0.408-1.272)	0.2581	16	0.827 (0.426-1.606)	0.5757
**ECOG-PS**	0	57	37	1(ref)		27	1(ref)	
	1 or more	16	11	0.901 (0.459-1.768)	0.7609	6	0.673 (0.279-1.623)	0.3779
**T stage**	T0, T1, T2	63	39	1(ref)		26	1(ref)	
	T3-T4	10	9	2.264 (1.087-4.717)	**0.029**	7	2.464 (1.067-5.693)	**0.0347**
**N stage**	N(-)	26	11	1(ref)		7	1(ref)	
	N(+)	47	37	2.597 (1.304-5.173)	**0.0066**	26	2.675 (1.152-6.21)	**0.022**
